# Internalizing and externalizing pathways to high-risk substance use and geographic location in Australian adolescents

**DOI:** 10.3389/fpsyg.2022.933488

**Published:** 2022-08-04

**Authors:** Bailey M. Willis, Phereby P. Kersh, Christy M. Buchanan, Veronica T. Cole

**Affiliations:** Department of Psychology, Wake Forest University, Winston-Salem, NC, United States

**Keywords:** internalizing and behavioral problems, substance use initiation, rurality, rural adolescents, survival mixture models

## Abstract

One specific instantiation of the storm-and-stress view of adolescence is the idea that “normal” adolescence involves high-risk substance use behaviors. However, although uptake of some substance use behaviors is more common during adolescence than other life stages, it is clear that not all adolescents engage in risky substance use—and among those who do, there is much variation in emotional, behavioral, and contextual precursors of this behavior. One such set of predictors forms the internalizing pathway to substance use disorder, whereby internalizing symptoms in childhood such as negative affect and anxiety set off a chain of consequences culminating in high-risk substance use in late adolescence. However, findings linking internalizing symptoms to substance use are mixed, and it is clear that this link varies across adolescents and contexts. One heretofore unanswered question is whether and how geographic location, specifically whether the adolescent lives in an urban or rural location, moderates this link. The current report is a secondary analysis of data from the Longitudinal Study of Australian Children (LSAC; *N* = 2,285), in which we examined the link between internalizing symptoms in childhood and initiation of substance use through age 19. Using a multiple event process survival mixture model (MEPSUM), we identified three trajectories of substance use initiation in adolescence: one (65.7% of the sample) characterized by near-complete abstinence until late adolescence, another (27.2%) by earlier initiation of alcohol, nicotine, and cannabis, and another (7.2%) by early initiation of these substances and later initiation of more hazardous drugs such as cocaine and methamphetamine. Although childhood externalizing symptoms increased the risk of being in the second or third class, internalizing symptoms decreased risk when rural and non-rural adolescents were considered together. Few effects of rurality were found, but the negative relationship between internalizing at age 10 and high-risk substance use was only observed among non-rural adolescents. This finding, which was inconsistent with our initial predictions that rurality might confer higher risk for substance use, instead suggests a potentially protective effect of internalizing symptoms for engagement in risky substance use which may differ based on an adolescent’s geographical context.

## Introduction

### Internalizing pathways to high-risk substance use in rural and non-rural adolescents

Substance use can have deleterious effects on adolescents, including the risk of progression to substance use disorder (SUD) in adulthood (McCabe et al., 2022). However, not all adolescents develop harmful forms of substance use, and not all contexts present equal levels of risk (Miech et al., 2022). Adolescence is a period of increased sensitivity to the uptake of health risk behaviors, due in part to increased risk taking during this period, but cognitive, affective, behavioral, and situational factors interact to produce increased levels of risk (Romer et al., 2017). The current study focuses on one potential pathway to substance use, through internalizing symptomatology throughout childhood and early adolescence, and examines the question of which environmental contexts are most conducive to this pathway unfolding. In particular, given recent evidence that adolescents in rural contexts may face different health risks than their urban counterparts (e.g., Jiang et al., 2016), we examine the question of whether and how internalizing and externalizing symptomatology interact with geographic location to produce different patterns of substance use initiation across adolescence. We first review pathways from negative affect to unhealthy substance use in adolescence from a developmental psychopathology framework, and then examine the evidence for different risk pathways among rural vs. urban youth.

### Negative affect and substance use in adolescence

A “storm and stress” framework, which has permeated both popular views of adolescents and academic theory and research (Montemayor et al., 1990; Buchanan and Bruton, 2016), highlights adolescence as a uniquely challenging time. In a critical review, Arnett (1999) argues that storm-and-stress theories are generally united by three domains in which adolescents struggle: negative mood or affect, conflict with parents, and engagement in risky behaviors. It is clear, however, that these issues do not manifest to the same extent, or interact with one another, in the same way across individuals and contexts (Arnett, 1999; Hollenstein and Lougheed, 2013). Other ways of conceptualizing risk in adolescence, such as the developmental psychopathology framework, or the “4T approach” (Hollenstein and Lougheed, 2013), allow for more individual differences based on person and environment. Researchers working from such perspectives have sought to identify configurations of traits and experiences that drive adolescents toward and away from healthy development (Sroufe and Rutter, 1984; Cicchetti, 2010).

Within the study of health risk behaviors such as risky substance use, one commonly identified pathway begins with externalizing symptoms in childhood. The externalizing pathway to substance use is marked by a pattern of dysregulated behavior beginning in childhood, which is associated with an escalating pattern of negative consequences throughout adolescence. These consequences, including academic underachievement, interpersonal difficulties, and association with delinquent peers, have been consistently associated with substance use both separately and in combination, providing robust evidence for this externalizing pathway (Zucker et al., 2011; Englund and Siebenbruner, 2012).

A smaller literature links childhood internalizing symptoms, such as anxiety and depressed mood, to the development of substance use disorder. Under this pathway, negative affect may both directly motivate substance use (i.e., self-medication) and indirectly predict it over time through mediators such as difficulty socializing and loneliness (Hussong et al., 2011). Evidence for this pathway is somewhat more complicated than for the externalizing pathway, with mixed results concerning the connection between internalizing symptoms and substance use after controlling for co-occurring externalizing symptoms (Colder et al., 2010; Hussong et al., 2017). Indeed, there is even some evidence that some internalizing symptoms, particularly anxiety, may be negatively related to substance use (Pardini et al., 2004, 2007; Fischer et al., 2012; Colder et al., 2013; Hersh et al., 2013; Scalco et al., 2014).

To the extent that internalizing symptoms in childhood are related to substance use during adolescence, it is clear that this association does not unfold in the same way for all adolescents. In particular, it can be hard to disentangle the effects of internalizing and externalizing symptoms, given the frequency with which internalizing and externalizing symptoms co-occur (Angold et al., 1999). Indeed, there is evidence for an “externalizing branch” of the internalizing pathway, whereby internalizing symptoms in childhood (e.g., depressed mood, shyness) predispose one to some externalizing behaviors later on (e.g., fighting, acting out in school), which more proximally predict unhealthy substance use. This pathway has been documented cross-culturally in adolescent samples from 12 different countries (Rothenberg et al., 2020). Additionally, there is evidence that aspects of an adolescent’s social context moderate the effects of internalizing symptoms on substance use both at a stable and time-varying level (Hussong et al., 2018, 2020). Low levels of social integration and high levels of coping motives may strengthen the tie between negative affect and substance use behavior (Cole et al., 2022). Affective and contextual moderators of this pathway need to be elucidated, in order to further understand which environments may be more conducive to this connection.

One of the main concerns with respect to substance use in adolescence is what constitutes high-risk substance use behavior. The Centers for Disease Control and Prevention defines it “as any use by adolescents of drugs with a high risk of adverse outcomes, such as injury, criminal justice involvement, school dropout, and loss of life” (CDC, 2022). However, this is a broad designation, which may refer to high quantity or frequency of substance use, using substances in dangerous situations (e.g., driving while intoxicated), early initiation of substance use, or the use of drugs such as opioids, cocaine, MDMA, or methamphetamine. Note that we refer to the category of drugs other than alcohol, nicotine, and cannabis as “more hazardous” drugs hereafter, on the basis of their low base rates in adolescence and perceived danger to personal and public health. We avoid the term “hard drugs” and acknowledge the imprecision in our own terminology (Janik et al., 2017).

There is evidence that earlier initiation of more hazardous drugs is linked to greater probability of developing substance use disorder in adulthood (Hser and Anglin, 2010). It is thus useful to examine an adolescent’s pattern of substance use initiation across multiple substances. This task may be accomplished using statistical models for the onset of multiple event processes. One such set of models is the multiple event process survival mixture (MEPSUM) model, which finds groups of individuals on the basis of multivariate patterns of hazard processes, in this case the initiation of the use of different substances (Dean et al., 2014). Multiple research teams have applied the MEPSUM model to substance use initiation, finding different groups of adolescents based on patterns of substance use onset (Dean et al., 2015; Richmond-Rakerd et al., 2016). A commonly found set of patterns includes at least one group of adolescents who initiate the use of commonly used substances perceived as less hazardous to one’s health, such as alcohol, nicotine and marijuana, in mid-to late adolescence; at least one group of adolescents who initiate the use of these substances early in adolescence and the use of more hazardous drugs (e.g., cocaine, opioids) later on; and one group of adolescents who abstain from substance use across the entire time period. Membership to higher-risk classes is generally associated with certain demographic predictors, such as being male, as well as Big Five personality traits such as higher levels of extraversion and lower levels of agreeableness and conscientiousness, and externalizing symptoms (Richmond-Rakerd et al., 2016).

### Risk mechanisms among rural youth

Within the United States, research supports variability in risk-taking activities and frequency across geographic locations (Fahs et al., 1999; Atav and Spencer, 2002). Despite the popular perception that urban environments are associated with increased risk, increasing attention to less explored areas suggests that some health risk behaviors may be more common among rural adolescents. Using secondary data spanning 7th through 11th grade in five Upstate New York school districts, Atav and Spencer (2002) examined differences between rural, suburban, and urban students in their frequency of various risk behaviors. Results indicated that rural adolescents from the sample were more likely to smoke, drink alcohol, and use other drugs than were their urban and suburban counterparts. Jiang et al. (2016) also found increased use of substances such as alcohol and nicotine among rural adolescents ages 12 to 19 in Arizona, but decreased use of more hazardous drugs such as cocaine and MDMA, relative to their urban counterparts of the same age range (Jiang et al., 2016). Riding in a car with a drunk driver as well as possessing stronger feelings of leniency toward drinking alcohol have also been found to be more common in rural areas within the United States (Smalley et al., 2019). There are many possible mechanisms that produce differences between urban and rural adolescents in health risk behaviors. For instance, relative to urban adolescents, rural adolescents may have access to fewer leisure activities, both structured (e.g., school activities, sports) and unstructured (e.g., shopping, going to events), yielding more time in which to engage in risky behaviors (Hardre et al., 2009).

There are also reasons to believe that the links among internalizing symptoms, externalizing symptoms, and substance use are different among rural youth. One reason is that rural geographic locations may present unique environmental stressors that contribute to the development of internalizing and externalizing behaviors (Curtis et al., 2011). Limited available research suggests that rural adolescents may be particularly vulnerable to developing anxiety and aggression, which are associated with internalizing and externalizing symptomatology, respectively (Smokowski et al., 2013). These behaviors have been linked to unique demands from their environments, such as stigma surrounding various health issues (Spoth et al., 2001; Smokowski et al., 2013). Perceived stigma pertaining to discussing or seeking mental health resources has been identified as a particular issue in rural areas of Australia in part because of the lack of anonymity in communities with lower population densities (Boyd et al., 2007; Brown et al., 2015). Compared to urban communities, where large population numbers may permit a sense of privacy for individuals with health issues, rural communities often increase the sense of social visibility of its inhabitants. Stronger stigmas together with higher social visibility may exacerbate feelings of anxiety or anger in rural adolescents as there are fewer resources available to address these related needs without risk of communal disapproval or negative labeling (Schroeder et al., 2020). Thus, rural adolescents may face greater difficulties than non-rural adolescents in receiving diagnoses or treatment for mental-health-related issues and may be expected to be self-reliant and push through their problems independently of outside support (Keller and Owens, 2021). There is also often a general lack of accessibility of health resources in rural areas, both in the United States and Australia, which may present as fewer mental health clinicians, longer wait times, and lower availability of appointments (Boyd et al., 2011; Black et al., 2012). It is possible that these contextual effects also moderate the link between some externalizing psychopathology and substance use, for instance the restricted social networks and higher pressures for conformity may further contribute to rural youth modeling disruptive behaviors of others, such as participating in substance use, to avoid ostracization from their broader community (Cotter and Smokowski, 2016). However, given the robust link between externalizing and substance use, moderating effects of rurality may be less salient. By contrast, internalizing appears to be linked to substance use only in some individuals and under some circumstances; thus, environmental effects are of particular interest here.

Despite the growing evidence that rural adolescents may be uniquely vulnerable to engaging in certain risk behaviors as well as developing internalizing and externalizing symptomology that is left untreated, the pathways at hand are certainly complex. Geographic setting is not expected to entirely explain these behaviors in adolescents, as adolescents in both rural and non-rural communities are at risk for negative outcomes from poverty (Crouch et al., 2019) or unstable home environments (Tucker et al., 2018), among an array of other potential sources. It is especially important to distinguish the stressors associated with rurality from those associated with poverty. While in some places individuals in rural locations may be of lower socioeconomic status than their urban counterparts, we are interested in the unique context that rural environments represent, beyond their potential to overlap with poverty. To do so, we account for the socioeconomic status of adolescents in the sample in addition to their environment. Thus, our study is designed to better understand the role that geographic setting plays in risk and psychopathology.

### The current study

In this study, we were interested in the affective and psychopathological precursors of substance use initiation patterns, as well as the question of whether these precursors differ across geographic classification. We hypothesized broadly that rural adolescents would be at increased risk for an enhanced link between childhood internalizing or externalizing psychopathology, on the one hand, and a pattern of adolescent substance use characterized by early use of substances such as alcohol and tobacco, on the other. To test this hypothesis, we applied a MEPSUM model to find different profiles of substance use initiation between the ages of 10 and 20. We assessed whether parent-and teacher-reported internalizing and externalizing symptoms in childhood predisposed adolescents to different patterns of substance use initiation, as well as whether this relationship differed in rural environments relative to urban ones.

These findings address the question of a storm and stress characterization of adolescence in at least two ways. First, to the extent that adolescent risk behaviors such as substance use are presaged by childhood patterns of mood or behavior, this would be inconsistent with the notion that adolescence is a discontinuous time of life, unique in its level of challenge and a result, primarily, of the biological changes underlying puberty and their sequelae. Second, a storm and stress characterization of adolescence predicts relatively universal change in maladaptive risk-taking at adolescence, although the timing of the changes might vary depending on the occurrence of biological changes or ubiquitous pressures for autonomy (Hall, 1904; Arnett, 1999; Buchanan and Bruton, 2016). If the developmental rates and trajectory of maladaptive substance use vary by childhood characteristics or context, such as geographical region, this would reduce the value of a storm and stress characterization, and be more consistent with a developmental psychopathology model, the latter of which puts more emphasis on individual differences, ranging from wellbeing to psychopathology, and is based on a combination of biological, individual, and contextual features rather than universal developmental changes (see also Hollenstein and Lougheed, 2013).

## Materials and methods

Data come from Growing Up in Australia: Longitudinal Study for Australian Children (LSAC), an ongoing nationally representative longitudinal study of Australian children beginning in 2004. There are two cohorts, one of which was first measured at birth and the other of which was first measured at kindergarten; we used the kindergarten cohort due to its ability to capture the entire range of adolescence. We examine data from waves 4 (age 10) through 8 (ages 18/19) based on the availability of our indicators of interest, described further below. Sampling and measurement procedures are described in detail elsewhere (Gray and Sanson, 2005).

### Participants

The sample (*N* = 2,285) consists of all participants with data on substance use initiation at wave 8, as well as internalizing and externalizing symptoms at wave 4; of those originally assessed at wave 1, 45.9% of participants were retained in our sample. Demographic information for the sample is shown in Table 1. As shown, race and ethnicity were broken down according to whether participants were Aboriginal/Torres Strait Islanders or not. Information about the primary language spoken at home was also provided, indicating that a relatively small percentage spoke languages other than English at home. A majority of participants came from urban environments, and roughly 14.7% of participants came from rural environments; categories of urbanicity/rurality were operationalized as described below.

**Table 1:** Demographic information for the analysis sample (*N* = 2,228).

**Percentage/mean (SD)**	
Male	50.33
Aboriginal/Torres Strait Islander	1.62
Language other than English spoken at home	6.66
Age at wave 8 in years	18.45 (0.503)
Socioeconomic status (SEP score) at wave 1	0.26 (0.97)
**Percentage initiated at any time over the study period**	
Alcohol	85.91
Cannabis	32.25
Hallucinogens	5.51
Inhalants	7.4
Cocaine	4.68
MDMA	11.95
Methamphetamine	2.31
Nicotine	44.72
Nonmedical use of prescription drugs	8.32

It is important to note that the 45.9% of the larger sample included in the current study were demographically different from the larger LSAC sample in a number of ways. Those who remained in the study at wave 8 were of higher socioeconomic status than those who did not participate in wave 8, *t*(4846.5) = −17.646, *p* < 0.001. Participants identifying as Aboriginal or Torres Strait Islander were less likely to be retained in the current sample, χ2 (1) = 52.099, *p* < 0.001, as were those who reported speaking a language other than English at home, χ2 (1) = 37.87, *p* < 0.001. However, rural participants were no more or less likely to be retained, χ^2^(1) = 2.8682, *p* = 0.09, nor were male participants, χ2 (1) = 0.49776, *p* = 0.4805.

### Procedure

Data collection is described in detail elsewhere (Gray and Sanson, 2005). Children born between March 1999 and February 2000 were randomly sampled within one of 311 randomly selected postcodes. Of the 18,800 families identified, 10,090 completed Wave 1 of the study; of these, 4,983 were in the kindergarten cohort studied here. After the initial assessment, follow-up assessments were conducted every 2 years, resulting in a total of nine possible waves of assessment at two-year intervals between the ages of four and 20 years. Each assessment entailed a battery of self-report measures to be answered by the study child’s primary parent, secondary parent if present, and the child’s educator. The self-report measures varied in length across waves, and consisted of a combination of face-to-face interviews, computer-assisted interviews, and self-report questionnaires. Informed consent was obtained from the child’s caregiver, and all procedures were approved by the Human Research Ethics Committee at the Australian Institute of Family Studies.

### Measures

#### Childhood internalizing and externalizing symptoms

Internalizing and externalizing symptoms were assessed at waves 2–8. Because we are interested in predicting substance use initiation across adolescence prospectively, we use internalizing and externalizing indicators from wave 4 (age 10). The questionnaire was completed by the child’s primary parent, secondary parent (if present), and teacher. For the current study, responses from the primary parent are used.

Internalizing and externalizing symptoms were assessed using the Emotional Problems and Conduct Problems scales of the Strengths and Difficulties Questionnaire (Goodman and Goodman, 2009), respectively. Each of these was a five-item scale. For each item respondents were instructed to describe the child’s behavior over the past 6 months using one of three response options: not true, somewhat true, or certainly true. Internal consistency was satisfactory for internalizing symptoms (α = 0.72) but suboptimal for externalizing symptoms (α = 0.58).

#### Substance use initiation

At wave 8, adolescents were asked whether they used a number of different substances over the course of their life. If they said yes, they were asked at what age they first initiated use of that substance. For each substance, participants were asked whether they had used the substance; if they had, they were asked at what age they first initiated use. First use of alcohol was defined as the first full drink a subject took, and first cigarette use was defined as the first time a participant smoked any portion of a cigarette, even just a puff. Additionally, some substance use categories were generated by collapsing two or more larger categories. For instance, cannabis use was based on a participant’s response to a question about whether they had ever used marijuana or synthetic cannabis. The following substances were assessed: alcohol, cigarettes, cannabis (collapsed across cannabis and synthetic marijuana); methamphetamine (collapsed across ice and other methamphetamine); inhalants; cocaine; hallucinogens; MDMA; non-medical prescription drug use (collapsed across tranquilizers, painkillers, and stimulants).

#### Urbanicity/rurality

The Australian Statistical Geographical Standard (ASGS) has four categories for Section of State (SOS): major urban (urban centers with population > 100,000); other urban (urban centers with population between 1,000 and 99,000); bounded localities (areas other than urban centers with population > 500); and rural balance (areas with population < 500). Following the ASGS designation, we coded the first two categories as urban and the latter two categories as rural (Australian Bureau of Statistics, 2016). This measure was administered at all waves; we use the measure from wave 4, when internalizing and externalizing symptoms were measured, here.

#### Socioeconomic status

Socioeconomic status was measured using a composite score calculated from each parent’s education level, income, and occupation status (Baker et al., 2017). Occupation status was coded using the Australian and New Zealand Standard Classification of Occupations (Australian Bureau of Statistics, 2009). As with urbanicity/rurality, we use the measure of socioeconomic status from wave 4.

### Analytic plan

Data management was conducted using R, and models were fit using M-plus (Muthén and Muthén, 2017). All mixture models were estimated using the expectation maximization (EM) algorithm, which uses all available information (i.e., pairwise deletion) under the assumption that data are missing at random. Complex sampling was used in the original data collection, and therefore all analyses were conducted using longitudinal survey weights.

A multiple event process survival mixture model (MEPSUM; Dean et al., 2014) was fit to each of the nine substance use initiation variables described above. Indicators were collapsed into 11 epochs corresponding to each year between ages 10 to 19. Data were coded such that each subject received a score of 0 for any epoch at which they had not initiated use of a given substance, and a score of 1 for the epoch in which they initiated the use of that substance, and all values afterward were set to missing. The number of classes was chosen after consulting the Bayesian and Akaike Information Criteria (BIC and AIC, respectively; Schwarz, 1978; Akaike et al., 1998), as well as the Vuong Lo Mendell Rubin (V-LMR; Lo et al., 2001) likelihood ratio test. Lower values of the BIC and AIC indicate a better balance of fit and parsimony. The V-LMR tests the null hypothesis that a *k*-class model fits better than a *k–*1-class model; the chosen solution is the last one for which the test yields a significant result. Although we would generally prefer to include the parametric bootstrap likelihood ratio test (BLRT), it is not able to be calculated when sampling weights are used. Note that we do not aim to reify these classes or imply that they are “real” in any sense—the main purpose of these classes is to generate data-driven classifications of substance use liability which can later be linked to predictors (i.e., a so-called indirect application of mixture modeling; Titterington et al., 1985).

After determining the number of classes, we estimated the relationship between these classes and the following set of predictors: sex, Aboriginal/Torres Strait Islander background (ATSI), a binary indicator of whether a language other than English was spoken at home (LOTE), rurality, internalizing symptoms, and externalizing symptoms. We fit one model containing solely main effects, and another which also included two two-way interaction terms: the interaction between internalizing symptoms and rurality, and the interaction between externalizing symptoms and rurality. Thus, to avoid the configuration of classes changing with each re-fitting of the model, three-step estimation was used (Vermunt, 2010; Asparouhov and Muthén, 2014). Thus, predictors did not affect the classes themselves.

## Results

Substance use initiation rates are shown in the lower half of Table 1. When modeling the hazard of substance initiation, most functions had to begin after age 10 due to the extremely low rates of substance use initiation at earlier time points. This includes cannabis, for which there was negligible use before age 11; inhalants, MDMA, and amphetamines, for which there was negligible use before age 13; and cocaine and hallucinogens, for which there was negligible use before age 14. As shown, the rate of substance use initiation was generally quite low overall, with only alcohol, nicotine, and cannabis being tried by more than 25% of the sample by age 19.

The number of classes was selected after consulting the fit indices shown in Table 2. As shown, the BIC and LMR likelihood ratio test both favored a 3-class solution, whereas the AIC continued to improve with increasing classes. Disagreement among different criteria is common in class enumeration, and the BIC has (along with the bootstrap likelihood ratio test, which could not be used here due to complex sampling) been identified as a more reliable criterion than the AIC (Nylund et al., 2007). We therefore proceed with a 3-class solution, favoring the BIC and V-LMR over the AIC.

**Table 2:** Fit statistics for classes with different numbers of solutions.

Num. classes	VLMR			
	AIC	BIC	χ^2^(73)	*p*-value
1	31133.1	31,546		
2	28007.8	28839.3	3271.299	<0.001
**3**	**27259.6**	**28509.6**	**894.279**	**0.0012**
4	27022.7	28691.2	391.784	0.2132

Akaike information criterion, Bayesian information criterion, and Vuong Lo Mendell Rubin test results for solutions with 1, 2, 3, and 4 classes. Note that the VLMR test comparing adjacent classes has 73 degrees of freedom, as a K-class solution has 73 more parameters than a K − 1-class solution in this case. Further note that a 5-class solution did not converge, and it is not considered further. The bolded text in table is in reference to our decision to utilize the 3-class solution, versus a 1, 2, or 4-class solution, to examine substance use initiation in our sample.

### Three-class solution

**Figure 1:**
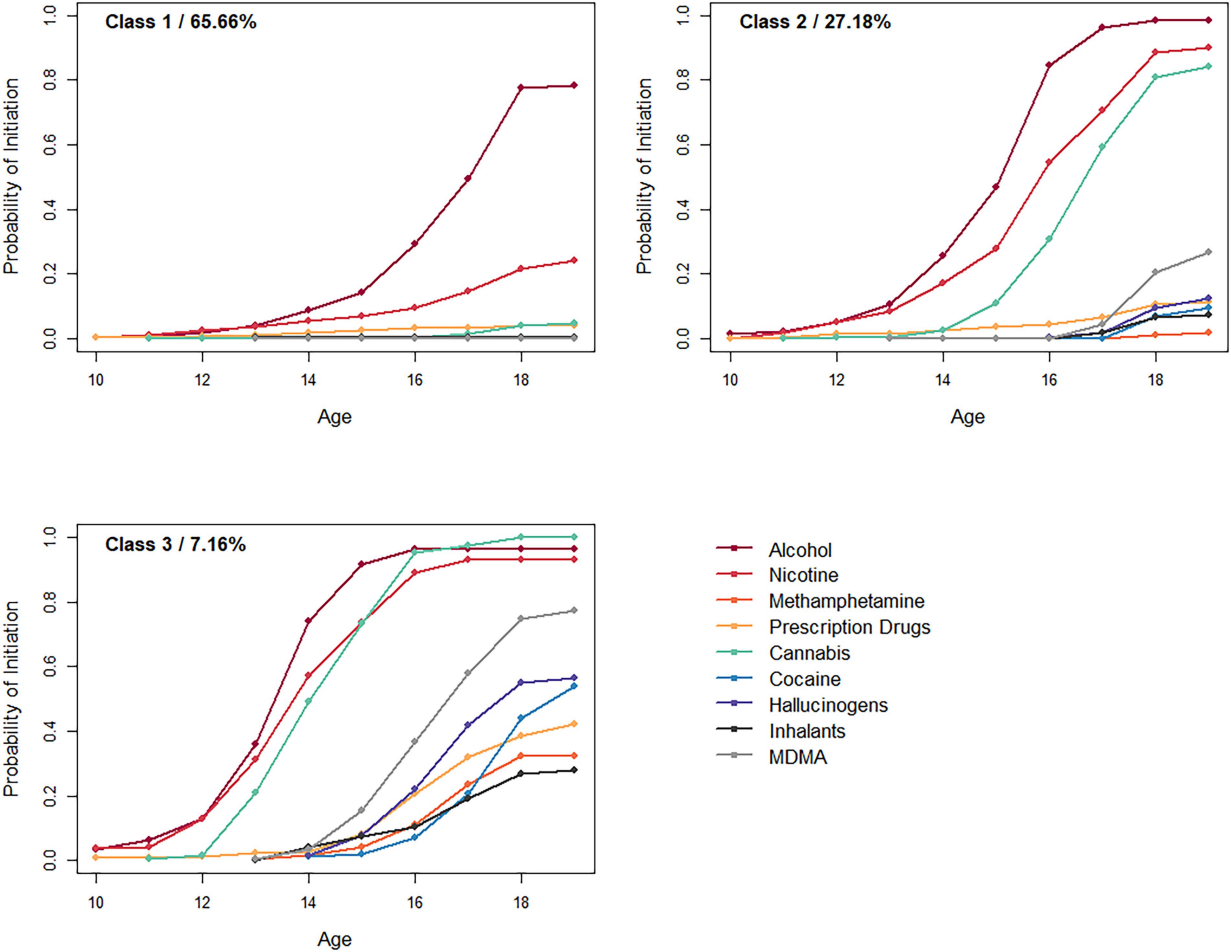
Probability of initiating use of each substance for each class.

Survival curves for substance use initiation under the 3-class solution are shown for each of the three classes in Figure 1.

Class 1, which describes roughly two thirds of the sample, was characterized by very low risk of substance use initiation across the study period. The risk of using any substances other than alcohol and nicotine was very low (< 5%) in this class. Both substances were initiated later than the other two classes; only at age 18 do members of this class have a more than 50% chance of having tried alcohol and a 20% chance of having tried nicotine. Note that, given that alcohol and tobacco are both legal to consume after age 18 in Australia, the typical member of this class engages exclusively in legal forms of substance use.

Class 2, which describes just over a quarter of the sample, is characterized by relatively early initiation of alcohol, tobacco, and cannabis use, with the initiation probability for these substances increasing rapidly between ages 13 and 16. Some more hazardous substance use is possible but relatively uncommon later on, with a roughly 25% chance of having tried MDMA by age 19 and a considerably lower (<15%) chance of having tried any other drug.

Finally, Class 3 was the smallest, describing roughly 7% of the sample. This class is characterized by early onset of alcohol, nicotine, and cannabis use, with risk beginning around age 12, culminating in an over 90% chance of having tried these substances by age 16. Later in adolescence, the risk for initiating MDMA, hallucinogens, and cocaine increases, with overall risk exceeding 50% by age 19. All substances have an over 25% initiation probability by age 19 in this class.

Taken together, the classes seem to increase somewhat continuously in overall substance use liability. They range from a majority of youth who do not engage in substance use except for late-onset experimentation with alcohol and nicotine (Class 1); to relatively early users of alcohol, nicotine, and tobacco who by and large do not engage in other substance use (Class 2); to those who initiate the use of alcohol, nicotine, and cannabis early, and move onto less normative, more hazardous forms of substance use later on (Class 3).

### Predictors of class membership

Table 3 shows results from two multinomial logistic regression models predicting latent class membership from demographic predictors (male, ATSI, SES, LOTE), rurality, internalizing and externalizing symptoms at age 10. The left half of the table shows a model with only main effects; the right half shows a model with two-way interactions between rurality and both internalizing and externalizing.

**Table 3:** Logistic regression parameters predicting class membership.

	Main effects model				Interaction model			
	Estimate		95% CI		Estimate		95% CI	
			Lower	Upper			Lower	Upper
**Class 2 vs. Class 1**								
Intercept	−0.584	**	−0.811	−0.357	−0.562	**	−0.799	−0.325
LOTE	−0.923	*	−1.699	−0.147	−0.93	*	−1.704	−0.156
SES	0.043		−0.098	0.184	0.041		−0.100	0.182
Male	−0.114		−0.371	0.143	−0.119		−0.378	0.140
ATSI	−1.299		−2.965	0.367	−1.288		−2.950	0.374
Internalizing	−0.155	**	−0.237	−0.073	−0.185	**	−0.277	−0.093
Externalizing	0.114	*	0.014	0.214	0.139	**	0.035	0.243
Rural	−0.024		−0.385	0.337	−0.126		−0.702	0.450
Internalizing x Rural					0.203	†	−0.011	0.417
Externalizing x Rural					−0.207		−0.536	0.122
**Class 3 vs. Class 1**								
Intercept	−2.521	**	−2.937	−2.105	−2.466	**	−2.891	−2.041
LOTE	−0.088		−0.921	0.745	−0.109		−0.936	0.718
SES	0.12		−0.111	0.351	0.115		−0.118	0.348
Male	0.132		−0.258	0.522	0.121		−0.267	0.509
ATSI	1.219	*	0.200	2.238	1.213	*	0.241	2.185
Internalizing	−0.123	*	−0.227	−0.019	−0.168	**	−0.293	−0.043
Externalizing	0.236	**	0.075	0.397	0.26	*	0.080	0.440
Rural	0.379		−0.199	0.957	0.153		−0.731	1.037
Internalizing x Rural					0.221	*	0.001	0.441
Externalizing x Rural					−0.145		−0.510	0.220
**Class 3 vs. Class 2**								
Intercept	−1.937	**	−2.388	−1.486	−1.903	**	−2.360	−1.446
LOTE	0.835		−0.278	1.948	0.822		−0.287	1.931
SES	0.078		−0.175	0.331	0.074		−0.179	0.327
Male	0.246		−0.195	0.687	0.24		−0.197	0.677
ATSI	2.519	**	0.820	4.218	2.501	**	0.821	4.181
Internalizing	0.033		−0.096	0.162	0.017		−0.136	0.170
Externalizing	0.122		−0.043	0.287	0.121		−0.063	0.305
Rural	0.403		−0.203	1.009	0.279		−0.652	1.210
Internalizing × Rural					0.018		−0.247	0.283
Externalizing × Rural					0.062		−0.338	0.462

LOTE, language other than English at home; ATSI, Aboriginal/Torres Strait Islander; SES, socioeconomic status.

† *p <* 0.1;

* *p* < 0.05;

** *p* < 0.01.

As shown, there were relatively few demographic predictors of class membership. Speaking a language other than English at home was associated with a higher probability of membership to Class 1 compared to Class 2 or Class 3. Identifying as Aboriginal/Torres Strait Islander was associated with an increased probability of being in Class 3 relative to Class 2 or Class 1. However, note that the percentage of ATSI participants was extremely low, suggesting that this finding is likely an artifact in our sample. Rurality did not predict class membership in any regression model.

**Figure 2:**
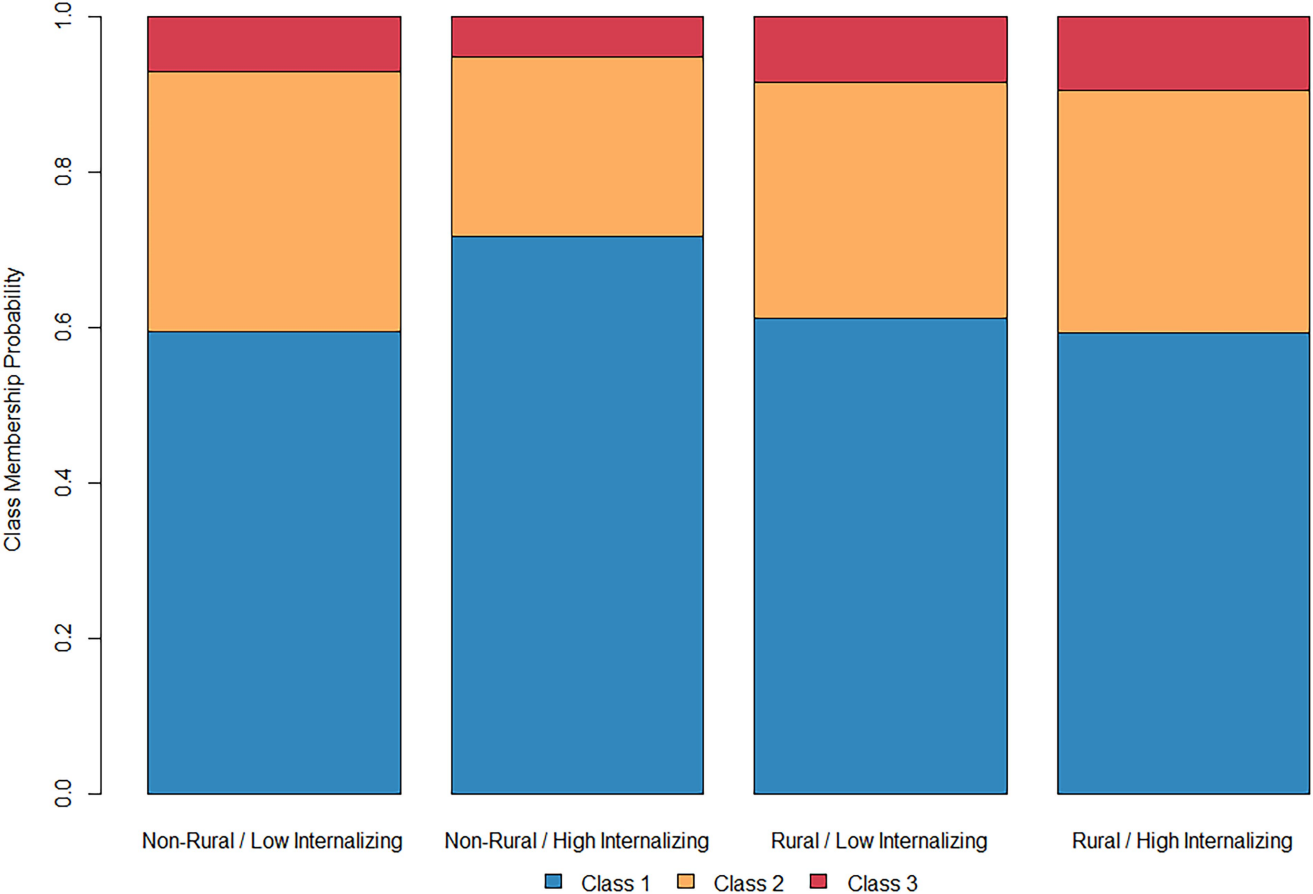
Class membership probability based on internalizing symptoms.

Considering rural and non-rural adolescents together in the main effects model, childhood externalizing symptoms were generally associated with increased overall substance use liability. Adolescents who had been high in externalizing at age 10 were more likely to be in Classes 2 or 3 than Class 1. By contrast, childhood internalizing symptoms appeared to exert the opposite effects; subjects high in internalizing were less likely to be in Classes 2 or 3 than Class 1. However, the model including two-way interactions complicates this finding, with a two-way interaction between rurality and internalizing symptoms in predicting membership to Class 1. Figure 2 shows predicted class membership probabilities for rural and non-rural participants at the 25th and 75th percentiles of internalizing (corresponding to a score of 0 and 3 respectively). Among non-rural adolescents, high levels of internalizing symptoms appear to be associated with an increased probability of being in Class 1 than either Classes 2 or 3, suggesting a somewhat lower probability of the use of alcohol, nicotine, and cannabis. As shown in Figure 1, this effect was not present among rural adolescents, with subjects high in internalizing symptoms actually being 1.8% less likely to be in Class 1 than their low-internalizing counterparts.

## Discussion

The current analysis investigated the links between different patterns of adolescent substance use initiation and childhood internalizing and externalizing symptoms among rural and non-rural adolescents. Using a subset of the Longitudinal Study of Australian Children dataset, we applied survival mixture modeling to substance use initiation data between ages 10–19 and found three classes, which ranged in severity from near-abstinence (Class 1); to early initiation of alcohol, nicotine, and cannabis with some potential use of more hazardous drugs later (Class 2); to initiation of more hazardous drug use before the end of adolescence (Class 3). Rurality itself did not affect the probability of class membership, giving no evidence of geographical region impacts on substance use initiation patterns in this sample. However, more childhood externalizing was linked to a higher probability of membership in the higher-risk substance use classes. Conversely, more childhood internalizing was linked to a lower probability of membership in these classes, such that adolescents who were identified as having greater levels of negative affect at age 10 were less likely to engage in risky substance use. There was also a significant interaction, such that the inverse relationship between internalizing symptoms and class membership was only apparent in non-rural adolescents. Taken together, these findings indicate a potentially protective effect of internalizing symptoms on substance use, one which may be moderated by geographic location.

Although the classes found here are better considered as a data reduction mechanism than meaningful categorical entities, it is important to consider the patterns of substance use initiation they represent in the context of those found in other samples. One benefit of this comparison is that it might provide insight into patterns of substance use beyond adolescence. When applied to substance use initiation data across age ranges spanning from adolescence through age 30, MEPSUM models have yielded solutions with four (Richmond-Rakerd et al., 2016, using the Add Health dataset) and six classes (Dean et al., 2015 using the NSDUH). Interestingly, the proportion of the sample in Class 1 here (comprising 65.7% of the sample) resembles a combination of two classes in each of these studies: the “relative abstainers” and “early adulthood users” found by Richmond-Rakerd et al., comprising a combined 61.1% of that sample; and “general abstainers” and “later soft drug use” found by Dean et al., comprising a combined 62.4% of that sample. It may be the case that the abstainers in Class 1 further separate into two patterns of substance use as they move through adulthood. Similarly, comparison to other adolescent samples helps to generalize to racial groups not explicitly considered here. For instance, when comparing patterns of substance use initiation among Black and White adolescent girls in the United States, Sartor et al. (2022) found differences between groups in the configuration of classes, but in both samples relative abstainers comprised just under 60% of the sample. We were not able to directly compare Black and White adolescents here, but this provides some indirect evidence of the generalizability of this pattern.

With respect to affective precursors of substance use, our findings are not inconsistent with the generally mixed findings concerning the pattern of relationships between internalizing pathways and substance use (Hussong et al., 2017). Some findings link elevated state-or trait-level depressive or anxious symptoms to more substance use, whereas others essentially find the opposite—that internalizing symptoms may actually be associated with decreased substance use liability (Pardini et al., 2004, 2007; Fischer et al., 2012; Colder et al., 2013; Hersh et al., 2013; Scalco et al., 2014). This study’s results are more consistent with the latter set of findings. More broadly, the finding that internalizing symptoms in childhood predict lower-risk patterns of substance use in adolescence is consistent with the hypothesis that internalizing symptoms exert a “braking effect” on deviant behavior (Masten et al., 2005; Burt et al., 2008). It has been hypothesized that, in some children and adolescents, some internalizing symptoms such as anxiety and shyness are reflective of high levels of behavioral inhibition, which may keep adolescents from seeking out dangerous contexts and engaging in risk behaviors. There is further evidence that internalizing symptoms may prevent risk behaviors by being socially limiting, as internalizing symptoms sometimes entail decreased social competence (Rogosch et al., 2010). Thus, it may be the case that internalizing symptoms are associated with exclusion from social contexts, such as parties or informal unsupervised social gatherings, in which risky substance use is more likely.

Under this logic, the interaction with rurality, whereby the increased probability of abstinence among adolescents with high levels of internalizing symptoms was only present in non-rural youth, may reflect differences between rural and urban youth in the structure of adolescent social ecosystems. Rural adolescents report perceiving less access to illicit substances (e.g., cannabis, MDMA) but greater access to legal substances (e.g., tobacco, alcohol) than their urban counterparts (Warren et al., 2015), a finding which is consistent with the prevalence of actual substance use among these groups (Jiang et al., 2016). It might be the case that differences in access between rural and non-rural adolescents are responsible for a different pattern of relationships between negative affect and substance use in these groups. However, this is only one possibility and considerably more research needs to be done before ascribing meaning to the interaction observed here.

In particular, it is important to exercise caution in interpreting the direction of this interaction, particularly in concluding that internalizing symptoms exert a protective effect only among non-rural adolescence. First, the percentage of adolescents in the sample from a rural area is relatively low, as less than 15% of the sample was classified as rural. In addition, the overall sample reported skewed levels of internalizing symptomatology; there were low levels of internalizing among the entire sample. The findings of this study may have yielded some preliminary evidence of internalizing pathways playing out differently between rural and urban adolescents, but the analyses should be replicated in a study with a larger percentage of rural adolescents and greater reports of internalizing psychopathology for further understanding of this phenomenon.

### Cultural considerations of the current study

It is important to consider the current study in the context of its geographic location. Although the LSAC represents a contemporary, nationally representative study of Australian youth, inferences based on the LSAC sample may not generalize beyond the population from which it was drawn, namely Australian youth between 2004 and 2021. Given that much research on rural adolescents has focused on U.S.-based and to a lesser extent Chinese samples, there may be features of the rural communities sampled in LSAC that are challenging to equate with prior findings. As an example, note that the use of opioids, one of the most frequently cited health issues in U.S. rural communities during this time period, was not even asked about in this study (except for the inclusion of heroin under the heading of “other drugs” in some questions). This may be due to the relatively low rates of opioid misuse and mortality in Australia relative to the U.S, and Canada (Brown and Morgan, 2019).

The study possesses a number of other differences in measurement from many U.S.-based samples, perhaps most notably the omission of a variable explicitly assessing race beyond ATSI designation. Although information about language spoken at home provides some insight into adolescents’ ethnic and cultural background, it is by no means a perfect proxy. Moreover, the information we do possess indicates that this sample may be quite ethnically homogenous, with only 1.62% of participants identifying as ATSI and only 6.66% speaking a language other than English at home. Another measurement issue arises with the definition of a rural area, which was defined here as anywhere other than an urban center. Though a population of 1,000 generally appears to be a lower bound on the definition of an urban center in the ASGS, some areas referred to “bounded localities,” which are generally suburban regions with a rural character, are also considered rural even if they had more than 1,000 residents. By contrast, the US Census Bureau defines a rural area as any falling outside an Urbanized Area (UA) or Urban Cluster (UC), effectively corresponding to a lower bound of 2,500 residents. Even beyond this difference, it is clear that social and cultural definitions of rurality contain information that is not captured by population size; we were not able to incorporate this level of nuance here.

A final difference between Australia and the United States which may bear on the results is that both alcohol and tobacco are licit for individuals 18 years or older (Australian Government Department of Health, 2022), whereas the legal age for both is 21 in the U.S. Thus, the use of these substances by adolescents may be perceived differently around the globe, as indicated by stricter or looser age requirements. This may also be related to the fact that lifetime alcohol use was more prevalent in the current sample (85.91%) than in American 12th grade students sampled during the same years (58.5%), despite the prevalence of nicotine use being similar and the prevalence of cannabis use being lower (Johnston et al., 2022). Moreover, given that one of the classes noted here (Class 1) was characterized by a nonzero probability of alcohol and tobacco use after age 18, it may be the case that members of this class were motivated to only engage in legal substance use. It is unclear whether the same results would be observed in the United States or other countries in which the legal drinking age is 21.

### Limitations of the current study

The current study was characterized by a number of limitations. First is the issue of generalizability. While the LSAC sample is nationally representative of the population of Australian adolescents, only 45.9% of this larger sample were included in the current analysis. As noted above, members of the current sample were of higher socioeconomic status, less likely to identify as ATSI, and less likely to speak a language other than English at home than members of the original LSAC sample who were not included. While survey weights may partially account for this discrepancy, it is important to recognize the limited generalizability of the subsample of LSAC included here.

Measurement represents another potential issue. The internal consistency of the internalizing and externalizing indicators (which was generally around 0.6) was suboptimal, which may have attenuated our estimates of the relationships among these constructs. Additionally, the relatively long period recall window, spanning ages 10 to 19, raises the possibility that participants were biased in their estimates of substance use initiation (Parra et al., 2003). A final critical caveat is that we investigated internalizing symptoms in childhood as opposed to adolescence. It may be the case that higher levels of internalizing symptoms in adolescence, reflecting greater levels of negative affect in that time period, are in some cases promotive of higher levels of substance use-related risk. Future research must investigate the contemporaneous links among these variables.

However, one clear conclusion emerges from research into internalizing pathways to substance use: risky substance use and negative affect are not experienced in the same way by all adolescents. Understanding the relationship between them, as well as person-and context-specific moderators of this relationship, will help us move toward understanding each adolescent’s unique experience.

### Implications for characterizing adolescents

The findings of this study highlight limitations of a storm and stress characterization of adolescence. It is true, and consistent with the theory, that risk taking in the form of substance use increased across the adolescent years. Nonetheless, a minority of adolescents (34%, in Classes 2 and 3) engaged in substance use, especially hazardous substance use, prior to the legal age. Although early or dangerous substance use in one-third of a group is of concern, accurately characterizing adolescents requires that we take note of the 66% who do not fit this profile. The largest class of adolescents (Class 1) initiated substance use relatively late in adolescence, with probabilities of initiating alcohol use exceeding 50% around age 17, and their substance use was limited to alcohol and nicotine, substances that are legal in Australia at 18 years of age. Thus, normative substance use seems more accurately characterized as age-appropriate and not especially hazardous. Furthermore, the fact that childhood behaviors, and to some extent geographic setting, predicted adolescent substance use trajectories is inconsistent with a storm and stress characterization emphasizing discontinuous changes in risk behavior that are primarily driven by biological change. We replicated earlier work showing that earlier initiation of and more dangerous substance use in adolescence is predicted by higher childhood externalizing (e.g., Racz et al., 2017; Krahé, 2020; Schmidt et al., 2021). Additionally, although childhood internalizing was less likely to predict adolescent substance use than was childhood externalizing, the results indicated that internalizing symptoms were potentially protective against substance use, an effect which was stronger in some settings than others. In other words, personal and contextual factors that promote participation in social settings increase the risk for hazardous substance use. In sum, we find evidence that adolescent substance use is at least somewhat continuous with childhood patterns of behavior mood or behavior, and modified by social context. These findings reduce the value of a storm and stress characterization and are more consistent with models of adolescent development that emphasize individual differences, contextual influences, and the interactions among these factors and biological influences (e.g., Cicchetti, 2010; Hollenstein and Lougheed, 2013).

## Data availability statement

The data analyzed in this study is subject to the following licenses/restrictions: users must apply for access through the Australian Institute of Family Studies at: https://growingupinaustralia.gov.au/data-and-documentation/accessing-lsac-data. Requests to access these datasets should be directed to https://growingupinaustralia.gov.au/data-and-documentation/accessing-lsac-data.

## Ethics statement

The studies involving human participants were reviewed and approved by Human Research Ethics Committee (HRECs), Australian Institute of Family Studies. Written informed consent to participate in this study was provided by the participants' legal guardian/next of kin.

## Author contributions

BW wrote the first draft of the Introduction and Discussion. VC wrote the first draft of the Materials and Methods and Results. PK found the data and organized all of the study variables. CB helped to generate the initial idea for the manuscript and edited drafts. All authors contributed to the article and approved the submitted version.

## Conflict of interest

The authors declare that the research was conducted in the absence of any commercial or financial relationships that could be construed as a potential conflict of interest.

## Publisher’s note

All claims expressed in this article are solely those of the authors and do not necessarily represent those of their affiliated organizations, or those of the publisher, the editors and the reviewers. Any product that may be evaluated in this article, or claim that may be made by its manufacturer, is not guaranteed or endorsed by the publisher.

## References

[ref1] Akaike H. Parzen E. Tanabe K. Kitagawa G. (1998). Selected Papers of Hirotugu Akaike. New York, NY: Springer Science & Business Media.

[ref2] Angold A. Costello E. J. Erkanli A. (1999). Comorbidity. J. Child Psychol. Psychiatry Allied Discip. 40, 57–87. doi: 10.1111/1469-7610.00424 10102726

[ref3] Arnett J. J. (1999). Adolescent storm and stress, reconsidered. Am. Psychol. 54, 317–326. doi: 10.1037/0003-066X.54.5.317, PMID: 10354802

[ref4] Asparouhov T. Muthén B. (2014). Auxiliary variables in mixture modeling: three-step approaches using M plus. Struct. Equ. Model. Multidiscip. J. 21, 329–341. doi: 10.1080/10705511.2014.915181

[ref5] Atav S. Spencer G. A. (2002). Health risk behaviors among adolescents attending rural, suburban, and urban schools: a comparative study. Fam. Community Health 25, 53–64. doi: 10.1097/00003727-200207000-00007, PMID: 12010115

[ref6] Australian Bureau of Statistics (2009). ANZSCO-Australian and New Zealand Standard Classification of Occupations. Canberra: Australian Bureau of Statistics.

[ref7] Australian Bureau of Statistics (2016). 1270.0.55.004—Australian Statistical Geography Standard (ASGS): Volume 4—Significant Urban Areas, Urban Centres and Localities, Section of State, July 2016. Canberra, Australia: Australian Bureau of Statistics.

[ref8] Australian Government Department of Health (2022). Alcohol Laws in Australia. Available at: https://www.health.gov.au/health-topics/alcohol/about-alcohol/alcohol-laws-in-australia (Accessed June 16, 2022).

[ref9] Baker K. Sipthorp M. Edwards B. (2017). A longitudinal Measure of Socioeconomic Position in LSAC. Australia: Australian Institute of Family Studies Melbourne.

[ref10] Black G. Roberts R. Li-Leng T. (2012). Depression in rural adolescents: relationships with gender and availability of mental health services. Rural Remote Health 12, 1–11. doi: 10.22605/RRH2092 22881194

[ref11] Boyd C. Francis K. Aisbett D. Newnham K. Sewell J. Dawes G.. (2007). Australian rural adolescents’ experiences of accessing psychological help for a mental health problem. Aust. J. Rural Health 15, 196–200. doi: 10.1111/j.1440-1584.2007.00884.x, PMID: 17542793

[ref12] Boyd C. Hayes L. Nurse S. Aisbett D. Francis K. Newnham K.. (2011). Preferences and intention of rural adolescents toward seeking help for mental health problems. Rural Remote Health 11, 122–134. doi: 10.22605/RRH1582 21319934

[ref13] Brown R. Morgan A. (2019). The opioid epidemic in North America: implications for Australia. Trends Issues Crime Crim. Justice 578, 1–15.

[ref14] Brown A. Rice S. Rickwood D. Parker A. (2015). Systematic review of barriers and facilitators to accessing and engaging with mental health care among at-risk young people. Asia Pac. Psychiatry 8, 3–22. doi: 10.1111/appy.12199, PMID: 26238088

[ref15] Buchanan C. M. Bruton J. L. (2016). “Storm and stress,” in Encyclopedia of Adolescence. ed. Levesque R. J. R. (NY: Springer).

[ref16] Burt K. B. Obradović J. Long J. D. Masten A. S. (2008). The interplay of social competence and psychopathology over 20 years: testing transactional and cascade models. Child Dev. 79, 359–374. doi: 10.1111/j.1467-8624.2007.01130.x, PMID: 18366428

[ref17] CDC (2022). Youth high-risk drug use. Available at: https://www.cdc.gov/healthyyouth/index.htm (Accessed June 16, 2022).

[ref18] Cicchetti D. (2010). Developmental Psychopathology. Hoboken, NJ: John Wiley & Sons, Inc.

[ref19] Colder C. R. Chassin L. Lee M. R. Villalta I. K. (2010). “Developmental perspectives: affect and adolescent substance use,” in Substance Abuse and Emotion ed. Kassel J. D. (Washington, DC: American Psychological Association).

[ref20] Colder C. R. Scalco M. Trucco E. M. Read J. P. Lengua L. J. Wieczorek W. F.. (2013). Prospective associations of internalizing and externalizing problems and their co-occurrence with early adolescent substance use. J. Abnorm. Child Psychol. 41, 667–677. doi: 10.1007/s10802-012-9701-0, PMID: 23242624 PMC3640685

[ref002] Cole V. Hussong A. McNeish D. Ennett S. Rothenberg A. Gottfredson N.. (2022). The role of social position within peer groups in distress-motivated smoking amongst adolescents. J. Stud. Alcohol Drugs 83, 420–429. doi: 10.15288/jsad.2022.83.420 35590183 PMC9134997

[ref21] Cotter K. Smokowski P. (2016). Perceived peer delinquency and externalizing behavior among rural youth: the role of descriptive norms and internalizing symptoms. J. Youth Adolesc. 45, 520–531. doi: 10.1007/s10964-015-0382-1, PMID: 26519368

[ref22] Crouch E. Radcliff E. Probst J. Bennett K. McKinney S. (2019). Rural-urban differences in adverse childhood experiences across a national sample of children. J. Rural Health 36, 55–64. doi: 10.1111/jrh.12366, PMID: 30938864

[ref23] Curtis A. C. Waters C. M. Brindis C. (2011). Rural adolescent health: the importance of prevention services in the rural community. J. Rural Health 27, 60–71. doi: 10.1111/j.1748-0361.2010.00319.x 21204973

[ref24] Dean D. O. Bauer D. J. Shanahan M. J. (2014). A discrete-time Multiple Event Process Survival Mixture (MEPSUM) model. Psychol. Methods 19, 251–264. doi: 10.1037/a0034281, PMID: 24079930 PMC4077031

[ref25] Dean D. O. Cole V. Bauer D. J. (2015). Delineating prototypical patterns of substance use initiations over time. Addiction 110, 585–594. doi: 10.1111/add.12816, PMID: 25429736

[ref26] Englund M. M. Siebenbruner J. (2012). Developmental pathways linking externalizing symptoms, internalizing symptoms, and academic competence to adolescent substance use. J. Adolesc. 35, 1123–1140. doi: 10.1016/j.adolescence.2012.03.004, PMID: 22465287 PMC3419769

[ref27] Fahs P. S. S. Smith B. E. Atav A. S. Britten M. X. Collins M. S. Morgan L. C. L.. (1999). Integrative research review of risk behaviors among adolescents in rural, suburban, and urban areas. J. Adolesc. Health 24, 230–243. doi: 10.1016/S1054-139X(98)00123-2 10227342

[ref28] Fischer J. A. Najman J. M. Williams G. M. Clavarino A. M. (2012). Childhood and adolescent psychopathology and subsequent tobacco smoking in young adults: findings from an Australian birth cohort. Addiction 107, 1669–1676. doi: 10.1111/j.1360-0443.2012.03846.x, PMID: 22340634

[ref29] Goodman A. Goodman R. (2009). Strengths and difficulties questionnaire as a dimensional measure of child mental health. J. Am. Acad. Child Adolesc. Psychiatry 48, 400–403. doi: 10.1097/CHI.0b013e3181985068, PMID: 19242383

[ref30] Gray M. Sanson A. (2005). Growing up in Australia: the longitudinal study of Australian children. Fam. Matters 72, 4–9.

[ref001] Hall G. S. (1904). Adolescence: Its Psychology and Its Relation to Physiology, Anthropology, Sociology, Sex, Crime, Religion, and Education (Vols I & II). Englewood Cliffs, NJ: Prentice-Hall.

[ref31] Hardre P. Sullivan D. Crowson H. M. (2009). Student characteristics and motivation in rural high schools. J. Res. Rural. Educ. 24, 1–19.

[ref32] Hersh J. Curry J. F. Becker S. J. (2013). The influence of comorbid depression and conduct disorder on MET/CBT treatment outcome for adolescent substance use disorders. Int. J. Cogn. Ther. 6, 325–341. doi: 10.1521/ijct.2013.6.4.325, PMID: 25197427 PMC4155521

[ref33] Hollenstein T. Lougheed J. P. (2013). Beyond storm and stress: typicality, transactions, timing, and temperament to account for adolescent change. Am. Psychol. 68, 444–454. doi: 10.1037/a0033586, PMID: 23915399

[ref34] Hser Y. I. Anglin M. D. (2010). “Addiction treatment and recovery careers,” in Addiction Recovery Management. eds. Kelly J. White’s W. (Totowa, NJ: Humana Press), 9–29.

[ref35] Hussong A. M. Ennett S. T. Cox M. J. Haroon M. (2017). A systematic review of the unique prospective association of negative affect symptoms and adolescent substance use controlling for externalizing symptoms. Psychol. Addict. Behav. 31, 137–147. doi: 10.1037/adb0000247, PMID: 28134539 PMC5344716

[ref36] Hussong A. M. Ennett S. T. McNeish D. M. Cole V. T. Gottfredson N. C. Rothenberg W. A.. (2020). Social network isolation mediates associations between risky symptoms and substance use in the high school transition. Dev. Psychopathol. 32, 615–630. doi: 10.1017/S095457941900049X, PMID: 31232267 PMC7011186

[ref37] Hussong A. M. Jones D. J. Stein G. L. Baucom D. H. Boeding S. (2011). An internalizing pathway to alcohol use and disorder. Psychol. Addict. Behav. 25, 390–404. doi: 10.1037/a0024519, PMID: 21823762 PMC3178003

[ref38] Hussong A. M. Rothenberg W. A. Smith R. K. Haroon M. (2018). Implications of Heterogeneity in Alcohol Use Disorders for Understanding Developmental Pathways and Prevention Programming. Oxford, England: Oxford University Press.

[ref39] Janik P. Kosticova M. Pecenak J. Turcek M. (2017). Categorization of psychoactive substances into “hard drugs” and “soft drugs”: a critical review of terminology used in current scientific literature. Am. J. Drug Alcohol Abuse 43, 636–646. doi: 10.1080/00952990.2017.1335736, PMID: 28650668

[ref40] Jiang G. Sun F. Marsiglia F. F. (2016). Rural–urban disparities in adolescent risky behaviors: a family social capital perspective. J. Community Psychol. 44, 1027–1039. doi: 10.1002/jcop.21825

[ref41] Johnston L. D. Miech R. A. O'Malley P. M. Bachman J. G. Schulenberg J. E. Patrick M. E. (2022). Monitoring the Future National Survey Results on Drug Use, 1975–2021: Overview, key Findings on Adolescent Drug Use. Ann Arbor, MI: Institute for Social Research, University of Michigan.

[ref42] Keller E. M. Owens G. P. (2021). Understanding help-seeking in rural counties: a serial mediation analysis. J. Clin. Psychol. 78, 857–876. doi: 10.1002/jclp.23260 34614200

[ref43] Krahé B. (2020). Risk factors for the development of aggressive behavior From middle childhood to adolescence: the interaction of person and environment. Curr. Dir. Psychol. Sci. 29, 333–339. doi: 10.1177/0963721420917721

[ref44] Lo Y. Mendell N. R. Rubin D. B. (2001). Testing the number of components in a normal mixture. Biometrika 88, 767–778. doi: 10.1093/biomet/88.3.767

[ref45] Masten A. S. Roisman G. I. Long J. D. Burt K. B. Obradović J. Riley J. R.. (2005). Developmental cascades: linking academic achievement and externalizing and internalizing symptoms over 20 years. Dev. Psychol. 41, 733–746. doi: 10.1037/0012-1649.41.5.733, PMID: 16173871

[ref46] McCabe S. E. Schulenberg J. E. Schepis T. S. McCabe V. V. Veliz P. T. (2022). Longitudinal analysis of substance use disorder symptom severity at age 18 years and substance use disorder in adulthood. JAMA Netw. Open 5:e225324. doi: 10.1001/jamanetworkopen.2022.5324, PMID: 35363270 PMC8976240

[ref47] Miech R. A. Johnston L. D. O’Malley P. M. Bachman J. G. Schulenberg J. E. Patrick M. E. (2022). Monitoring the Future national Survey Results on Drug use, 1975–2021: Volume I, Secondary school Students. Ann Arbor: Institute for Social Research, The University of Michigan.

[ref48] Montemayor R. Adams G. R. Gullotta T. P. (eds.) (1990). From Childhood to Adolescence: A transitional period? Newbury Park, CA: Sage Publications.

[ref49] Muthén B. Muthén L. (2017). Mplus. London, England: Chapman and Hall/CRC.

[ref50] Nylund K. L. Asparouhov T. Muthén B. O. (2007). Deciding on the number of classes in latent class analysis and growth mixture modeling: a Monte Carlo simulation study. Struct. Equ. Model. Multidiscip. J. 14, 535–569. doi: 10.1080/10705510701575396

[ref51] Pardini D. Lochman J. Wells K. (2004). Negative emotions and alcohol use initiation in high-risk boys: the moderating effect of good inhibitory control. J. Abnorm. Child Psychol. 32, 505–518. doi: 10.1023/B:JACP.0000037780.22849.23, PMID: 15500030

[ref52] Pardini D. White H. R. Stouthamer-Loeber M. (2007). Early adolescent psychopathology as a predictor of alcohol use disorders by young adulthood. Drug Alcohol Depend. 88, S38–S49. doi: 10.1016/j.drugalcdep.2006.12.014, PMID: 17257781 PMC2034413

[ref53] Parra G. R. O'Neill S. E. Sher K. J. (2003). Reliability of self-reported age of substance involvement onset. Psychol. Addict. Behav. 17, 211–218. doi: 10.1037/0893-164X.17.3.211, PMID: 14498815

[ref55] Racz S. J. Putnick D. L. Suwalsky J. T. D. Hendricks C. Bornstein M. H. (2017). Cognitive abilities, social adaptation, and externalizing behavior problems in childhood and adolescence: specific cascade effects across development. J. Youth Adolesc. 46, 1688–1701. doi: 10.1007/s10964-016-0602-3, PMID: 27815666 PMC5822001

[ref56] Richmond-Rakerd L. S. Fleming K. A. Slutske W. S. (2016). Investigating progression in substance use initiation using a discrete-time multiple event process survival mixture (MEPSUM) approach. Clin. Psychol. Sci. 4, 167–182. doi: 10.1177/2167702615587457, PMID: 27127730 PMC4844227

[ref57] Rogosch F. A. Oshri A. Cicchetti D. (2010). From child maltreatment to adolescent cannabis abuse and dependence: a developmental cascade model. Dev. Psychopathol. 22, 883–897. doi: 10.1017/S0954579410000520, PMID: 20883588 PMC3030981

[ref58] Romer D. Reyna V. Satterthwaite T. (2017). Beyond stereotypes of adolescent risk taking: placing the adolescent brain in developmental context. Dev. Cogn. Neurosci. 27, 19–34. doi: 10.1016/j.dcn.2017.07.007, PMID: 28777995 PMC5626621

[ref59] Rothenberg W. A. Lansford J. E. Chang L. Deater-Deckard K. Di Giunta L. Dodge K. A.. (2020). Examining the internalizing pathway to substance use frequency in 10 cultural groups. Addict. Behav. 102:106214. doi: 10.1016/j.addbeh.2019.106214, PMID: 31809879 PMC6961811

[ref60] Sartor C. E. Ye F. Simon P. Hipwell A. E. Chung T. (2022). Cross-substance patterns of alcohol, cigarette, and cannabis use initiation in Black and White adolescent girls. Prev. Med. 156:106979. doi: 10.1016/j.ypmed.2022.106979, PMID: 35124100 PMC8922285

[ref61] Scalco M. D. Colder C. R. Hawk L. W. Jr. Read J. P. Wieczorek W. F. Lengua L. J. (2014). Internalizing and externalizing problem behavior and early adolescent substance use: a test of a latent variable interaction and conditional indirect effects. Psychol. Addict. Behav. 28, 828–840. doi: 10.1037/a0035805, PMID: 25134030 PMC4165783

[ref62] Schmidt A. T. Camins J. S. Henderson C. E. Christensen M. R. Magyar M. S. Crosby J. W.. (2021). Identifying the contributions of maternal factors and early childhood externalizing behavior on adolescent delinquency. Child Psychiatry Hum. Dev. 52, 544–553. doi: 10.1007/s10578-020-01040-2, PMID: 32779072

[ref63] Schroeder S. Tan C. Urlacher B. Heitkamp T. (2020). The role of rural and urban geography and gender in community stigma around mental illness. Health Educ. Behav. 48, 63–73. doi: 10.1177/1090198120974963, PMID: 33218261

[ref64] Schwarz G. (1978). Estimating the dimension of a model. Ann. Stat. 6, 461–464.

[ref65] Smalley K. B. Warren J. C. Tarasenko Y. N. Barefoot K. N. (2019). The impact of rurality on likelihood of drunk driving and riding with a driver under the influence among high school students. J. Rural Health 35, 354–361. doi: 10.1111/jrh.12321, PMID: 30160320

[ref66] Smokowski P. R. Cotter K. L. Robertson C. I. Guo S. (2013). Anxiety and aggression in rural youth: baseline results from the rural adaptation project. Child Psychiatry Hum. Dev. 44, 479–492. doi: 10.1007/s10578-012-0342-x, PMID: 23108500

[ref67] Spoth R. Goldberg C. Neppl T. Trudeau L. Ramisetty-Mikler S. (2001). Rural–urban differences in the distribution of parent-reported risk factors for substance use among young adolescents. J. Subst. Abuse 13, 609–623. doi: 10.1016/S0899-3289(01)00091-8, PMID: 11775086

[ref68] Sroufe L. A. Rutter M. (1984). The domain of developmental psychopathology. Child Dev. 55, 17–29. doi: 10.2307/1129832 6705619

[ref69] Titterington D. M. Afm S. Smith A. F. Makov U. E. (1985). Statistical Analysis of finite Mixture Distributions. *Vol.* 198. Hoboken, NJ: John Wiley & Sons Incorporated.

[ref70] Tucker C. Sharp E. Van Gundy K. Rebellon C. (2018). Household chaos, hostile parenting, and adolescents’ well-being two years later. J. Child Fam. Stud. 27, 3701–3708. doi: 10.1007/s10826-018-1998-x

[ref71] Vermunt J. K. (2010). Latent class modeling with covariates: two improved three-step approaches. Polit. Anal. 18, 450–469. doi: 10.1093/pan/mpq025

[ref72] Warren J. C. Smalley K. B. Barefoot K. N. (2015). Perceived ease of access to alcohol, tobacco, and other substances in rural and urban US students. Rural Remote Health 15:3397. doi: 10.22605/RRH3397, PMID: 26518286 PMC4727394

[ref73] Zucker R. A. Heitzeg M. M. Nigg J. T. (2011). Parsing the undercontrol–disinhibition pathway to substance use disorders: a multilevel developmental problem. Child Dev. Perspect. 5, 248–255. doi: 10.1111/j.1750-8606.2011.00172.x, PMID: 22116786 PMC3221325

